# Curcumin Has Beneficial Effects on Lysosomal Alpha-Galactosidase: Potential Implications for the Cure of Fabry Disease

**DOI:** 10.3390/ijms24021095

**Published:** 2023-01-06

**Authors:** Maria Monticelli, Bruno Hay Mele, Mariateresa Allocca, Ludovica Liguori, Jan Lukas, Maria Chiara Monti, Elva Morretta, Maria Vittoria Cubellis, Giuseppina Andreotti

**Affiliations:** 1 Department of Biology, University of Napoli “Federico II”, Complesso Universitario Monte Sant’Angelo, Via Cinthia, 80126 Napoli, Italy; 2 Institute of Biomolecular Chemistry ICB, CNR, Via Campi Flegrei 34, 80078 Pozzuoli, Italy; 3 Department of Environmental, Biological, and Pharmaceutical Sciences and Technologies (DiSTABiF), University of Campania “Luigi Vanvitelli”, Via Vivaldi 43, 81100 Caserta, Italy; 4 Translational Neurodegeneration Section “Albrecht-Kossel”, Department of Neurology, University Medical Center Rostock, 18147 Rostock, Germany; 5 Center for Transdisciplinary Neurosciences Rostock (CTNR), University Medical Center Rostock, 18147 Rostock, Germany; 6 Department of Pharmacy, University of Salerno, Via Giovanni Paolo II 132, 84084 Fisciano, Italy

**Keywords:** curcumin, nutraceutical, drug repositioning, Fabry disease, lysosomal storage diseases, pharmacological chaperone, AGAL

## Abstract

Fabry disease is a lysosomal storage disease caused by mutations in the *GLA* gene that encodes alpha-galactosidase (AGAL). The disease causes abnormal globotriaosylceramide (Gb3) storage in the lysosomes. Variants responsible for the genotypic spectrum of Fabry disease include mutations that abolish enzymatic activity and those that cause protein instability. The latter can be successfully treated with small molecules that either bind and stabilize AGAL or indirectly improve its cellular activity. This paper describes the first attempt to reposition curcumin, a nutraceutical, to treat Fabry disease. We tested the efficacy of curcumin in a cell model and found an improvement in AGAL activity for 80% of the tested mutant genotypes (four out of five tested). The fold-increase was dependent on the mutant and ranged from 1.4 to 2.2. We produced evidence that supports a co-chaperone role for curcumin when administered with AGAL pharmacological chaperones (1-deoxygalactonojirimycin and galactose). The combined treatment with curcumin and either pharmacological chaperone was beneficial for four out of five tested mutants and showed fold-increases ranging from 1.1 to 2.3 for DGJ and from 1.1 to 2.8 for galactose. Finally, we tested a long-term treatment on one mutant (L300F) and detected an improvement in Gb3 clearance and lysosomal markers (LAMP-1 and GAA). Altogether, our findings confirmed the necessity of personalized therapies for Fabry patients and paved the way to further studies and trials of treatments for Fabry disease.

## 1. Introduction

In the last 20 years, the keyword “curcumin” increased its presence from 0 to 0.2% of the total publication volume in biochemistry, medicine, and pharmacology. Roughly 2000 papers on curcumin were published in 2021 (as represented by English articles found on Scopus belonging to the BIOC, MEDI, and PHARM subject areas). Curcumin is a turmeric-derived compound, the interest in which in Western medicine is based on its historical usage in Chinese medicine. In fact, turmeric (*Curcuma longa* L.) has a long tradition as a treatment for different illnesses that range from oxidative stress-related pathogenesis to anorexia [1,2,3,4].

The long history of turmeric usage allowed the U.S. Food and Drug Administration (FDA) to classify curcumin as Generally Recognized As Safe (GRAS). The wide spectrum of its biological activities justifies the interest of the scientific community in this unique and interesting molecule [5]. Many pathologies such as cancer and chronic diseases, diabetes, Alzheimer’s disease, autoimmune diseases, and rare diseases have been discovered to benefit from curcumin treatment, often in combination with other drugs [6,7,8,9,10,11,12,13,14,15,16,17,18]. Its pleiotropic effects are related to its actions in different pathways that include the PI3K, Akt, mTOR, ERK5, AP-1, TGF-β, Wnt, β-catenin, Shh, PAK1, Rac1, STAT3, PPARγ, EBPα, NLRP3 inflammasome, p38MAPK, Nrf2, Notch-1, AMPK, TLR-4, and MyD-88 pathways. In addition, curcumin modulates autophagy and endoplasmic reticulum (ER) stress [19]. The main issue associated with the clinical use of curcumin is its poor bioavailability. Given the high biomedical interest, great effort is being put in identifying new ways to improve this aspect using many different types of formulations [5,20,21,22].

Fabry disease (FD) is an LSD characterized by abnormal globotriaosylceramide (Gb3) storage in the lysosomes. Such abnormality is caused by the deficiency of the enzyme acidic alpha-galactosidase (AGAL) [23,24,25,26]. This protein, which is encoded by the *GLA* gene, is synthesized in precursor form, imported into the ER for glycosylation, and then targeted to the lysosomes to catalyze glycosphingolipid hydrolysis [27]. Whereas mutations in the *GLA* gene that either prevent synthesis or produce truncated variants of AGAL give rise to the most severe genotypes, missense mutations can have a more variable effect based on the location of the mutation. For example, the mutation could fall within the active site or cause a substitution that prevents folding, thereby strongly impacting protein function. Alternatively, the mutation could occur at flexible exposed sites and destabilize the protein. Variants belonging to the second subgroup are intrinsically active but are cleared by the quality-control system. In these cases, the total AGAL activity is insufficient for the correct clearance of Gb3. In addition to the effect on AGAL activity, it was recently demonstrated that missense mutations caused ER stress [28]. It is worth noting that none of the many (>2000) mutations in *GLA* associated with FD were prevalent among the patients [29,30,31,32,33].

Depending on the genotype, two therapeutic options are available for FD patients. In the most severe cases—patients unable to produce AGAL—enzyme replacement therapy (ERT) is mandatory. In all the other cases where at least a functional protein is still synthesized, the patient can undergo pharmacological chaperone therapy (PCT) [30,34]. In PCT, the patient is given pharmacological chaperones (PCs), which are small molecules that specifically bind and stabilize proteins. While PCT and ERT are both lifelong measures, the former is less expensive, eligible for oral administration, and reaches the central nervous system (CNS). Furthermore, while all FD patients can benefit from ERT regardless of the genotype severity, the quality of life of less severe cases was significantly improved if treated with PCT. Thus, a massive effort was put in place to predict responsive mutations and test PCs on hundreds of genotypes using either fibroblasts or leucocytes derived from patients or transiently transfected COS or HEK cells as cellular models [32,35,36]. In addition, particular attention has been focused on the clinical identification of putatively responsive patients via many tools and educational resources for students and physicians [37,38,39].

At the time of writing, the only PC approved for FD was 1-deoxygalactonojirimycin (DGJ) [40,41,42]. However, this molecule has the undesirable characteristic of being a substrate analog for AGAL, meaning it can inhibit the enzyme, particularly because it also binds the protein at an acidic pH [43,44]. Therefore, the FDA requires a precise posology to balance the inhibiting and chaperoning activities. Such a balance is achieved by using an intermittent administration regimen [42,45,46,47].

This paper describes the first attempt to use a nutraceutical (curcumin) to treat FD. We tested the hypothesis that it could be used to enhance AGAL activity in FD cells both alone or in synergy with the two PCs (DGJ or galactose).

## 2. Results and Discussion

**Figure 1:**
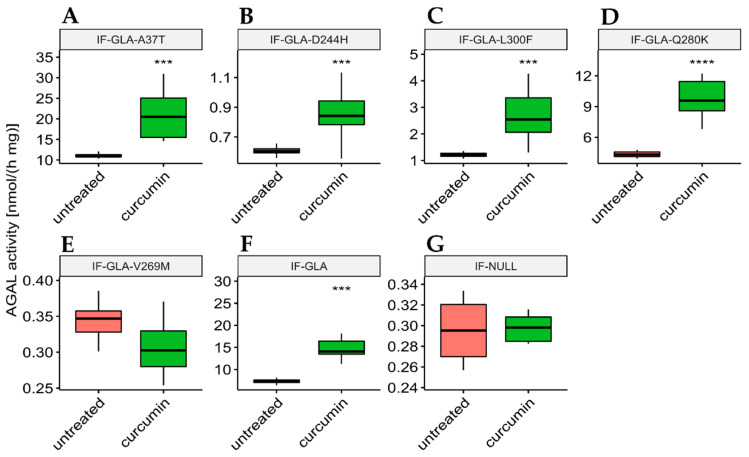
Curcumin treatment enhanced AGAL activity. IF-GLA and IF-GLA-MUTs were treated with 20 µM curcumin for 48 h. The AGAL-specific activity was then tested on cell protein extracts. Wild-type cells and four mutants showed a significant AGAL improvement upon curcumin treatment (two-tailed unpaired *t*-test, *** = *p* < 1 × 10^−3^, **** = *p* < 1 × 10^−4^). (**A**) IF-GLA-A37T: *n* = 9, *p*. 1.85 × 10^−4^; (**B**) IF-GLA-D244H: *n* = 12, *p*. 1.02 × 10^−4^ (**C**) IF-GLA-L300F: *n* = 10, *p*. 1.43 × 10^−4^; (**D**) IF-GLA-Q280K: *n* = 6, *p*. 9.68 × 10^−5^; (**E**) IF-GLA-V269M: *n* = 6, *p*. 1.08 × 10^−1^ (**F**) IF-GLA: *n* = 7, *p*. 5.72 × 10^−4^. IF-NULL cells were used as a negative control (**G**).

**Figure 2:**
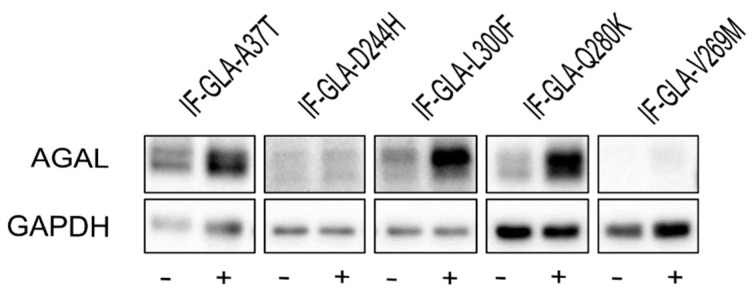
Curcumin treatment increased AGAL. IF-GLA and IF-GLA-MUTs were treated with 20 µM curcumin for 48 h. AGAL was visualized via immunoblotting on cell protein extracts. The figure shows IF-GLA-MUTs treated with (+) or without (−) 20 µM curcumin.

In this study, we tested the effect of curcumin treatment on AGAL activity over a panel of five mutants; specifically: c.109G>A (p.Ala37Thr, A37T), c.730G>C (p.Asp244His, D244H), c.898C>T (p.Leu300Phe, L300F), c.838C>A (p.Gln280Lys, Q280K), and c.805G>A (p.Val269Met, V269M). In particular, p.A37T causes the atypical renal-dominant phenotype [48], while p.D244H, p.Q280K, and p.V269M are related to the classic phenotype [49,50,51]; no information was given on the phenotype associated with p.L300F [52]. Immortalized fibroblasts transfected with individual pCMV6-AC plasmids carrying the five *GLA* mutants (IF-GLA-MUTs), immortalized fibroblasts transfected with the pCMV6-AC plasmid carrying wt-*GLA* (IF-GLA), and immortalized fibroblasts transfected with the empty vector (IF-NULL) obtained as described in [28] were treated with 20 μM curcumin or with no drug for 48 h. The time and dose of curcumin were selected based on the data available in literature, particularly those regarding cytotoxicity on fibroblasts [53,54,55]. After treatment, the AGAL-specific activity was tested on cell protein extracts. As shown in Figure 1, the wild type and all mutants except one (IF-GLA-V269M) showed a significant AGAL improvement upon curcumin treatment. An immunoblot reflected the enzyme activity results (Figure 2). The activity fold-increase varied between 1.4 (IF-GLA-D244H) and 2.2 (IF-GLA-L300F and IF-GLA-Q280K).

**Figure 3:**
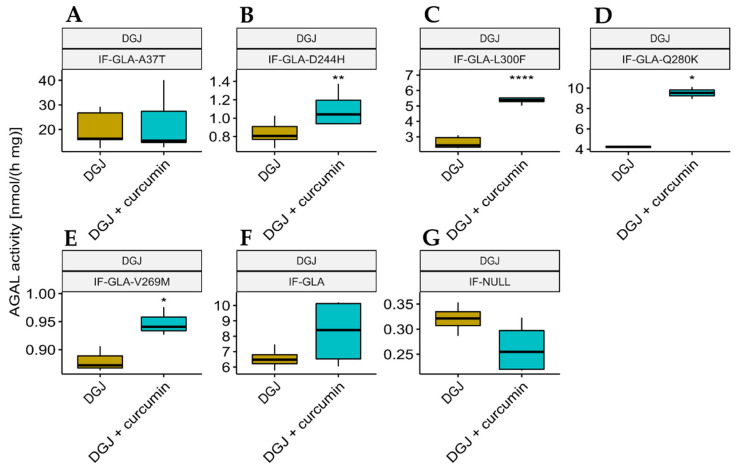
Combined treatment with DGJ and curcumin enhanced AGAL activity. IF-GLA and IF-GLA-MUTs were treated for 48 h with 10 µM DGJ in the presence or absence of 20 µM curcumin. The AGAL-specific activity was then tested on cell protein extracts. The presence of curcumin improved AGAL stabilization induced by DGJ in four out of the five tested mutants (two-tailed unpaired *t*-test, * = *p* < 5 × 10^−2^, ** = *p* < 1 × 10^−2^, **** = *p* < 1 × 10^−4^). (**A**) IF-GLA-A37T: *n* = 5, *p*. 8.01 × 10^−1^; (**B**) IF-GLA-D244H: *n* = 9, *p*. 2.64 × 10^−3^; (**C**) IF-GLA-L300F: *n* = 6, *p*. 5.03 × 10^−5^; (**D**) IF-GLA-Q280K: *n* = 2, *p*. 1.22 × 10^−2^; (**E**) IF-GLA-V269M: *n* = 3, *p*. 2.66 × 10^−2^; (**F**) IF-GLA: *n* = 4, *p*. 1.89 × 10^−1^. IF-NULL cells were used as a negative control (**G**).

**Figure 4:**
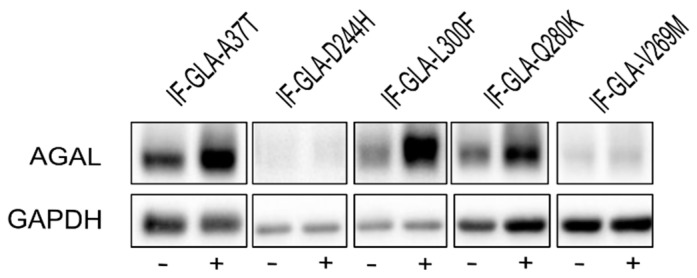
Combined treatment with DGJ and curcumin treatment increased AGAL. IF-GLA and IF-GLA-MUTs were treated for 48 h with 10 µM DGJ in the presence or absence of 20 µM curcumin. AGAL was visualized via immunoblotting on cell protein extracts. The figure shows IF-GLA-MUTs treated with 10 µM DGJ in the presence (+) or the absence (−) of 20 µM curcumin.

We also explored the potential of curcumin as a PC enhancer; i.e., a molecule able to strengthen the chaperoning effect of PCs. Cells were treated for 48 h with 10 μM DGJ either alone or in combination with 20 μM curcumin, and the protein extracts were analyzed. Figure 3 shows that the presence of curcumin improved AGAL stabilization induced by DGJ in four out of the five tested mutants (L300F, D244H, Q280K, and V269M) in a fold-change range of 1.1 (IF-GLA-V269M) to 2.3 (IF-GLA-L300F). As shown in Figure 4, immunoblots reflected the results.

**Figure 5:**
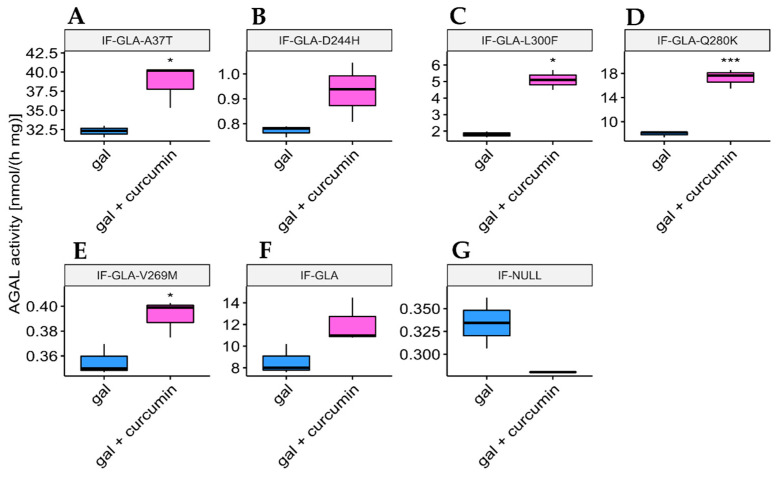
AGAL activity increased upon combined treatment with galactose and curcumin. IF-GLA and IF-GLA-MUTs were treated for 48 h with 100 mM galactose in the presence or the absence of 20 µM curcumin. The AGAL-specific activity was then tested on cell protein extracts. The effect of galactose potentiation was appreciated in four out of the five tested mutants (two-tailed unpaired *t*-test, * = *p* < 5 × 10^−2^, *** = *p* < 1 × 10^−3^). (**A**) IF-GLA-A37T: *n* = 3, *p*. 2.06 × 10^−2^; (**B**) IF-GLA-D244H: *n* = 3, *p*. 8.61 × 10^−2^; (**C**) IF-GLA-L300F: *n* = 2, *p*. 3.41 × 10^−2^; (**D**) IF-GLA-Q280K: *n* = 3, *p*. 6.31 × 10^−4^; (**E**) IF-GLA-V269M: *n* = 3, *p*. 3.1 × 10^−2^; (**F**) IF-GLA: *n* = 3, *p*. 7.36 × 10^−2^. IF-NULL cells were used as a negative control (**G**).

**Figure 6:**
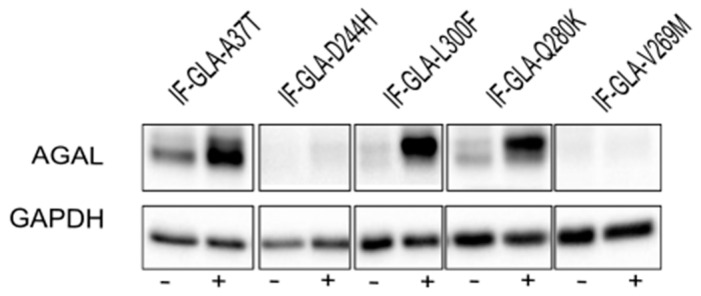
Combined treatment with galactose and curcumin treatment increased AGAL. IF-GLA and IF-GLA-MUTs were treated for 48 h with 100 mM galactose in the presence or absence of 20 µM curcumin. AGAL was visualized via immunoblotting on cell protein extracts. The figure shows IF-GLA-MUTs treated with 100 mM galactose in the presence (+) or the absence (−) of 20 µM curcumin.

In addition to DGJ, which is approved for therapeutical use, galactose is a low-affinity PC for AGAL, thus it requires a high dosage for patients to receive a beneficial effect. Investigation of the potential use of galactose supplementation showed very promising results for other rare diseases (mainly congenital disorders of glycosylation) [56,57,58,59,60]. Therefore, we tested whether the presence of curcumin could improve its stabilizing effect. We analyzed protein extracts derived from cells treated for 48 h with 100 mM galactose either alone or in combination with 20 μM curcumin. As Figure 5 shows, galactose potentiation benefited four out of the five tested mutants in a fold-change range of 1.1 (IF-GLA-V269M) to 2.8 (IF-GLA-L300F). Immunoblots reflecting the enzyme activity results are shown in Figure 6. Interestingly, different mutants showed different behaviors when moving to the combined therapies with PCs. For example, mutant IF-GLA-A37T did not show DGJ potentiation upon curcumin treatment (Figure 3). On the contrary, its activity improved upon combined treatment with galactose and curcumin with respect to galactose monotherapy (Figure 5). The opposite behavior was detected for IF-GLA-D244H, which was responsive to the combination of DGJ and curcumin but not to galactose and curcumin. These results were particularly interesting in a disease that requires personalized therapies.

**Figure 7:**
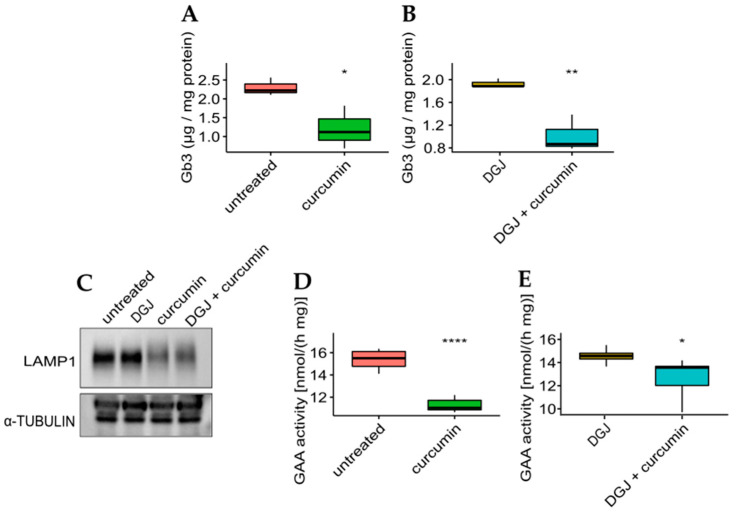
Curcumin treatment improved Gb3 clearance and lysosomal markers. (**A**,**B**) IF-GLA-L300F cells were treated for 50 days with drug administration once a week. At the end of the treatment (day 50), cells were collected and lipids were extracted through a methanol:chloroform:water protocol. Lipid content was measured via LC-MS/MS. (**A**) IF-GLA-L300F treated with 20 µM curcumin or with no drug showed improved Gb3 clearance upon curcumin treatment (two-tailed unpaired *t*-test, * = *p* < 5 × 10^−2^, *n* = 3, *p*. 3.79 × 10^−2^). (**B**) IF-GLA-L300F treated with 10 µM DGJ and 20 µM curcumin showed Gb3 clearance improved with respect to DGJ monotherapy (two-tailed unpaired *t*-test; ** = *p* < 1 × 10^−2^, *n* = 3, *p*. 8.96 × 10^−3^). (**C**–**E**) IF-GLA-L300F cells were treated for 15 days with 10 µM DGJ, 20 µM curcumin, or with both drugs. At the end of the treatment, the immunoblot showed a reduction in the levels of lysosome-associated membrane glycoprotein 1 (LAMP-1) upon curcumin treatment with or without DGJ (**C**). In addition, a reduction in GAA activity was determined via an enzyme activity assay both in monotherapy (**D**) (two-tailed unpaired *t*-test, **** = *p* < 1 × 10^−4^, *n* = 6, *p*. 3.95 × 10^−6^) or in combined therapy with DGJ (**E**) (two-tailed unpaired *t*-test * = *p* < 5 × 10^−2^, *n* = 6, *p*. 3.3 × 10^−2^).

The effectiveness of curcumin was then evaluated concerning the phenotypic response to the monotherapy or combined therapy on the mutant with the highest fold-increase for all the treatments. The IF-GLA-L300F mutant was treated for up to 50 days with drug administration every seven days. At the end of the long-term treatments, cells were lysed, lipid extraction was accomplished following a described protocol, and Gb3 content was evaluated via LC-MS/MS [61,62]. As shown in Figure 7, Gb3 clearance was significantly improved upon curcumin treatment both in monotherapy (panel A) or combined therapy with DGJ (panel B). The Gb3 quantity in treated cells (Figure 7A,B) was comparable to that of IF-GLA (Supplementary Figure S1A).

Jehn et al. [63] recently described alterations in the lysosomal pathway in the context of FD. In particular, they reported an increased expression in lysosomal hydrolases that was potentially due to Gb3 accumulation in lysosomes. Pereira et al. [64] described higher levels of lysosome-associated membrane protein 1 (LAMP-1) in FD lymphocytes compared to healthy controls, and we were able to confirm this upregulation in our FD cell model (Supplementary Figure S1B). The IF-NULL cells also showed higher levels of acidic α-glucosidase (GAA) than IF-GLA (Supplementary Figure S1C).

To evaluate the effect of curcumin treatment on potential lysosomal biomarkers, the IF-L300F cell line was treated with curcumin with or without DGJ. As shown in Figure 7, curcumin treatment resulted in a reduction in LAMP-1 levels (panel C), which had been previously described upon ERT treatment [64,65]. In addition, GAA activity is reduced upon curcumin treatment, in curcumin monotherapy (panel D), or in combined therapy (panel E). These results highlighted the beneficial effect of curcumin treatment on the FD cell model.

Curcumin has been described to disrupt Hsp90’s molecular function [66,67,68]. The inhibition mechanism is not fully understood; one hypothesis is the disruption of p210 bcr/abl with the Hsp90/p23 complex [66]. Sang et al. demonstrated the major role of Hsp90 in the curcumin-mediated protective effect in an Alzheimer’s disease cell model [69]. In particular, silencing Hsp90 significantly attenuated the rescuing effect of curcumin, while Hsp90 overexpression facilitated its effect. Jehn et al. previously demonstrated that AGAL rescue in an FD model had a prominent effect on Hsp90 expression, thus suggesting a role of the molecular chaperon in AGAL folding [63]. We hypothesized that Hsp90 has a prominent role in mediating curcumin effects in FD, thereby allowing AGAL precursor stabilization. Its action on the exosome/microvesicle secretion pathway has also been observed, which suggests its potential role in the treatments of LSDs [70]. Further studies will be needed both to explore the mechanism of action of curcumin in FD and to focus on a wider panel of mutations and precisely analyze their responsiveness to the mono- or combined therapies. We are aware that curcumin preparations might contain curcuminoid contaminants and would be better described as curcuminoid extracts.

FD patients show multiple clinical phenotypes. The classical form of FD usually presents symptoms such as neuropathic pain, cornea verticillata, and angiokeratoma, as well as long-term manifestations such as hypertrophic cardiomyopathy, cardiac rhythm disturbances, progressive renal failure, and strokes. In nonclassical FD, also known as late-onset or atypical FD, patients have residual enzyme activity and lower levels of the deacetylated substrate. This disease form is characterized by milder manifestations that affect just a single organ [71]. However, nonclassical FD patients may experience the same long-term effects as in the classical form [72].

The central role of dysregulated autophagy in LSDs was proposed many years ago [73], and its predominance in FD was recently highlighted. In fact, autophagy is among the mechanisms that underlie the FD phenotype together with overall lysosomal dysfunction, lipid dysmetabolism, and inflammation [74]. Tens of different biological effects of curcumin have been described over the years, and more applications are still being recorded yearly [22]. These mainly concern human health and mostly refer to the improvement of common pathological conditions that include both mild or severe conditions. Molecular mechanisms have already been studied [5,21]. It is worth noting that the beneficial effects of curcumin on cardiac and kidney function have been associated with the direct action of the molecule on autophagy and inflammatory pathways [75,76,77,78,79,80,81,82].

The beneficial effects of curcumin also have been described in cases of rare diseases such as Niemann–Pick type C disease (NPC) [17], neuronal ceroid lipofuscinosis (NCL) [83], and Tay–Sachs disease [18]. It is of utmost importance that for NPC, a triple combination therapy using miglustat, curcumin, and ibuprofen was investigated that resulted in a greater neuroprotective benefit compared with single and dual therapies [84].

Herein, we did demonstrate in an FD cell model that both curcumin or curcumin combined with DGJ produced an improvement in the phenotype of FD. Heart failure and renal involvement are significant issues for FD patients. Thus, the action of curcumin on different molecular mechanisms that leads to a general improvement might underlie its possible benefits in a large cohort of patients with different phenotypes.

These results represent a preliminary study, and further research will be needed to fully address the feasibility of moving from in vitro models to patients. Nevertheless, our results are promising because they open the field to using curcumin for both classic and non-classic FD phenotypes. Of course, the number of genotypes analyzed will need to be widened, and the effects of curcumin will have to be tested in different models (mainly patients’ fibroblasts or lymphoblasts carrying different mutations). In addition, the timing and dosage of curcumin will have to be defined.

## 3. Materials and Methods

### 3.1. Materials

The RPMI, fetal bovine serum, and reagents for cell cultures were purchased from Euroclone (Milan, Italy).

The curcumin was from BDH Chemicals Ltd., Poole, England (product number: 20031).

The DGJ was from Cayman Chemicals (Ann Arbor, Michigan, USA, product number: 17179). The cell lysis buffer (Roche product number: 4719956001), 4-methylumbelliferyl α-D-galactopyranoside (product number: M7633), 4-methylumbelliferone (product number: M1381), N-acetyl-D-galactosamine (product number: A2795), and lactosylsphingosine (product number: 42137) were purchased from Merck (Milan, Italy). The 4-methylumbelliferyl-α-D-glucopyranoside (product number: 69591) was from Biochemika (Merck, Milan, Italy). The Bradford dye (product number: 5000205), 30% acrylamide/bis soln. 37.5:1 (product number: 1610159), Clarity Western ECL (product number: 1705060), PVDF membranes (product number: 1620175), and Precision Plus Protein All Blue Standards (product number: 1610373) were purchased from Bio-Rad (Milan, Italy).

The Alpha-Galactosidase Polyclonal Antibody (product number: PA5-27349) and GAPDH Loading Control Monoclonal Antibody (product number: MA5-15738) were purchased from ThermoFisher Scientific (Milan, Italy). The lysosome-associated membrane glycoprotein 1 antibody (product number: H4A3, DHSB) was a kind gift from the Telethon Institute of Genetics and Medicine (TIGEM). All of the materials were used without further purification.

### 3.2. Cell Cultures

Immortalized patient-derived fibroblasts carrying a large deletion in *GLA* exons 3 and 4 were obtained from the Telethon Biobank and stably transfected as described in Monticelli et al. [62]. Cells were cultured in RPMI 1640 medium supplemented with 10% fetal bovine serum, 2 mM glutamine, 0.5 mg/mL penicillin, 0.5 mg/mL streptomycin, and non-essential amino acids at 37 °C in 5% humidified CO_2_. Geneticin 0.1 mg/mL was used to maintain the selection. Treatments with drugs were performed in the absence of geneticin. Drugs were dissolved in dimethyl sulfoxide, which represented the control for the untreated samples.

### 3.3. Enzymatic Activity Assays

Fibroblasts from a 90% confluent 20 cm^2^ plate were collected in Roche M cOmplete lysis buffer then centrifuged at 14,000× *g* for 10 min. An AGAL enzymatic activity assay was performed as described in [85] with the modifications described in Monticelli et al. [62]. Briefly, 40 µg of protein extracts were incubated with 0.4 mM 4-methylumbelliferyl-galactopyranoside and 8.7 mM N-acetylgalactosamine at 37 °C for 60 min in McIlvaine pH 4.4 buffer. A GAA enzymatic activity assay was performed similarly via incubation of a total of 20 µg of protein with 1.3 mM 4-methylumbelliferyl-α-D-glucopyranoside at 37 °C for 60 min in McIlvaine buffer pH 4.4. The reactions were stopped by the addition of GlyNaOH 1 M pH 10.5, and the fluorescence at 365/460 nm ex/em was read. 4-Methylumbelliferone was used for the calibration curves.

### 3.4. Gb3 Extraction

The extraction was accomplished according to the protocol outlined by Bligh and Dyer [61] with a few modifications as described in [62]. Briefly, cell pellets were lysed via resuspension in water and freezing–thawing, the soluble proteins were measured, and then lactosylsphingosine was added as an internal standard (2.5 ng of standard/µg of protein). Lipid extraction was performed with chloroform, methanol, water, and hydrochloric acid up to a final condition of (1:1:1:0.05) added in the following order: (i) chloroform/methanol (1:2); (ii) HCl; (iii) chloroform; (iv) water. Centrifuging (1500× *g*, 45 min at 20 °C) allowed us to obtain upper and lower phases. The samples were eventually dried under nitrogen and then analyzed via liquid chromatography–tandem mass spectrometry. For the UPLC-MS/MS analysis of Gb3, see Monticelli et al. [62].

### 3.5. Miscellaneous

The protein concentration was determined using the Bradford method with BSA as the standard [86]. Immunoblotting was performed under standard conditions as given in [40]. The data handling, analysis, and visualization were performed using the R environment for statistical computing (v4.2.1) with the tidyverse collection of packages (v1.3.1) [87,88] for the data handling and the rstatix (v0.7.0) [89] and ggpubr (v0.4.0) [90] packages for the statistical analysis and visualization, respectively. We performed unpaired two-tailed *t*-tests using the rstatix::*t*-test() function. All of the experiments were performed at least in biological duplicate; each biological duplicate was analyzed at least in technical duplicate. Biological replicates were considered in the statistical analysis.

## 4. Conclusions

This paper presented the results of a pilot study aimed at improving therapeutic approaches to FD. An AGAL increase in cells is highly beneficial for FD patients regardless of the mechanism leading to this effect. This is demonstrated in the approved therapies (ERT and PCT) [91] based on very different approaches but with the same outcome. We explored the possibility of obtaining this effect in an FD cell model upon curcumin treatment by analyzing the improvement in AGAL activity and the AGAL increase via immunoblotting.

The wide spectrum of biological activities makes curcumin a very unique and interesting molecule in the biomedical field [5]. For this reason, there is a large amount of literature in the field that will certainly benefit the Fabry community. In particular, data on the absorption, distribution, metabolism, and excretion (ADME) of curcumin have been collected over several decades that shows that curcumin bioavailability poses a severe limitation to its application for therapeutic purposes. To overcome this issue, different formulations to enhance curcumin bioavailability have already been widely investigated and clinical outcomes are also available. These research outputs include the combination of curcumin with adjuvants, the discovery of its structural analogues, its nanoformulations, and its inclusion in liposomes or phospholipid complexes [20,21,22]. A recent review on the topic that was published in 2021 highlighted the urgency of clinical trials to analyze the efficiency of nanoforms of curcumin as well as its derivative analogues [21]. Remarkably, one bio-enhanced derivative of curcumin was been successfully tested in a cell model of a rare variant form of Gaucher disease (GD) caused by mutations in the prosaposin gene (*PSAP*) [92].

Drug development and approval are multi-step processes that require significant investments and have poor chances of successful results [93]. These limitations imply a significant difficulty in improving new drugs for rare diseases. In this context, drug repositioning—using previously approved drugs for new therapeutic purposes—is a strategy for overcoming some problems related to de novo drug discovery. Repositioning reduces the time “between bench and bedside” while keeping research-related costs low. Most importantly, it reduces the risk of failure from more than 95% to around 45% [94,95,96,97,98,99]. Therefore, drug repurposing is a highly recommended practice in the scientific community, particularly for rare diseases [93].

The results described herein represent a case of nutraceutical repositioning for FD that pave the way to improving personalized therapies. We believe that this approach would be highly beneficial for patients, increase the advantages of PCT, and lead to the broadening of the target audience treatable with oral therapy.

Our proposal for FD is even more promising when considering that combined therapeutic approaches for rare diseases have been proposed [100,101,102,103,104] and that most importantly, one of them was recently approved by the FDA for the treatment of cystic fibrosis [105].

In conclusion, we believe that our results pave a new way toward the improvement of FD therapies, notwithstanding the limitations of our study and the further research needed for the translation to a medical approach.

## Data Availability

The data presented in this study are available in the article and supplementary material.

## Supplementary Materials

The following supporting information can be downloaded at: https://www.mdpi.com/article/10.3390/ijms24021095/s1.

## Abbreviations

AGAL: acidic alpha-galactosidase; CNS: central nervous system; DGJ: 1-deoxygalactonojirimycin; ER: endoplasmic reticulum; ERT: enzyme replacement therapy; FD: Fabry disease; FDA: Food and Drug Administration; GAA: acidic alpha-glucosidase; Gb3: globotriaosylceramide; IF-GLA: immortalized fibroblasts transfected with individual pCMV6-AC plasmid carrying wt-*GLA*; IF-GLA-MUTs: immortalized fibroblasts transfected with individual pCMV6-AC plasmids carrying *GLA* mutants; IF-NULL: immortalized fibroblasts transfected with the empty vector; LAMP-1: lysosomal-associated membrane protein 1; LSD: lysosomal storage disease; NPC: Niemann–Pick type C disease; PC: pharmacological chaperone; PCT: pharmacological chaperone therapy.
