# Supplementary figures and images for: Curcumin Has Beneficial Effects on Lysosomal Alpha-Galactosidase: Potential Implications for the Cure of Fabry Disease
Source: Int J Mol Sci. 2023 Jan 6;24(2):1095. doi: 10.3390/ijms24021095 (PMC9863837; doi:10.3390/ijms24021095)

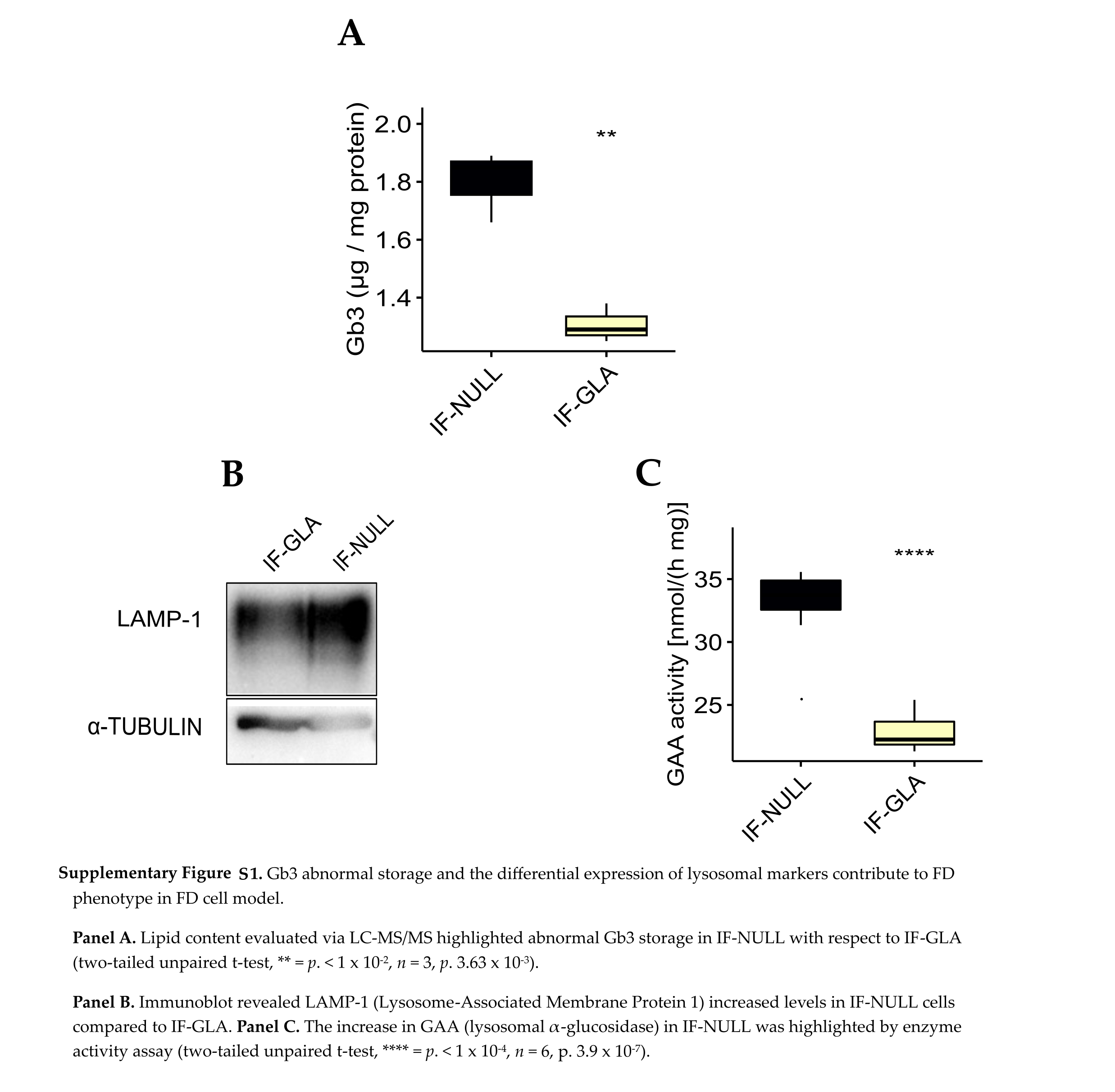

Supplement: Supplementary file 1 [file ijms-24-01095-s001.zip › ijms-2068192-supplementary.png]
